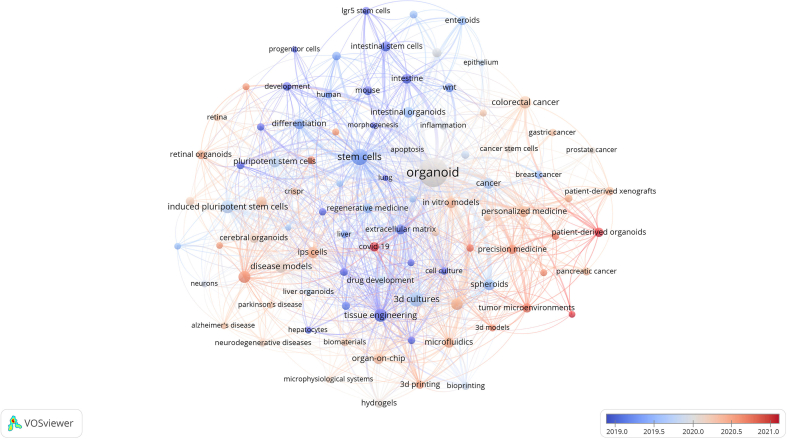# Current trends and research topics regarding organoids: A bibliometric analysis of global research from 2000 to 2023

**DOI:** 10.1016/j.heliyon.2024.e32965

**Published:** 2024-06-15

**Authors:** Yantong Wan, Jianan Ding, Zixuan Jia, Yinghao Hong, Guijie Tian, Shuqian Zheng, Pinfei Pan, Jieyan Wang, Hui Liang

**Affiliations:** a Department of Urology, People's Hospital of Longhua, Shenzhen, Guangdong, 518109, China; b Guangdong Provincial Key Laboratory of Proteomics, Department of Pathophysiology, School of Basic Medical Sciences, Southern Medical University, Guangzhou, China; c School of Basic Medical Sciences, Southern Medical University Guangzhou, China; d School of Chinese Medicine, Southern Medical University, Guangzhou, China; e School of Laboratory Medicine and Biotechnology, Southern Medical University Guangzhou, China; f The First School of Clinical Medicine, Southern Medical University, Guangzhou, China

**Keywords:** CiteSpace, VOSviewer, Organoids, Cancer, Drug discovery, Personalized medicine

## Abstract

The use of animal models for biological experiments is no longer sufficient for research related to human life and disease. The development of organ tissues has replaced animal models by mimicking the structure, function, development and homeostasis of natural organs. This provides more opportunities to study human diseases such as cancer, infectious diseases and genetic disorders. In this study, bibliometric methods were used to analyze organoid-related articles published over the last 20+ years to identify emerging trends and frontiers in organoid research. A total of 13,143 articles from 4125 institutions in 86 countries or regions were included in the analysis. The number of papers increased steadily over the 20-year period. The United States was the leading country in terms of number of papers and citations. Harvard Medical School had the highest number of papers published. Keyword analysis revealed research trends and focus areas such as organ tissues, stem cells, 3D culture and tissue engineering. In conclusion, this study used bibliometric and visualization methods to explore the field of organoid research and found that organ tissues are receiving increasing attention in areas such as cancer, drug discovery, personalized medicine, genetic disease modelling and gene repair, making them a current research hotspot and a future research trend.

## 1 Introduction

Organoid is an emerging technology that has only developed in the last two decades. It is possible to differentiate almost any cell type using adult stem cells or pluripotent stem cells. Through three-dimensional culture, they can form structures similar to whole organs, called organoids, whose microstructure is indistinguishable from that of natural organs in vivo [1,2]. Previously, two-dimensional (2D) cell lines were used extensively in various diseases, but they are limited by genetic drift and culture conditions, which make it challenging to generate new cell lines rapidly and reliably from patient samples. In addition, cell lines grown in two-dimensional (2D) monolayers are generally not representative of the original structure [3]. To overcome these drawbacks, researchers have created a three-dimensional (3D) tissue development culture system called organoids [4]. And during the last decade, a variety of organ types have been successfully designed for culture through 3D culturing of human ES and iPS cells, including skin, colon, retina, etc. [[5], [6], [7]]. And even functional tissues and organs such as functional spleen organoids, organoids that mimics the structure and function of the lungs and organoids simulating the beating of a heart have been realized [[8], [9], [10]]. Organoids have been used for studies that require direct experimentation on human tissue and need to be able to access imaging within complex multicellular systems [11]. For example, Porotto et al. [12] in order to reproduce the process of viral infection in respiratory patients, cultured embryonic stem cell lines embedded in Matrigel into organoids and reproduced them with parainfluenza infection. In parallel, these organoids are used not only in infectious diseases but also in treating various types of cancer. Due to the retention of features of their origin, both tumor xenografts and tumor organ models have become critical preclinical models for cancer research [13]. At the same time, organoid technology brings great hope for personalized medicine by creating individual tumor organs and implementing high-throughput drug screening, pairing drug sensitivity information with detailed genomic information to stratify individual patients and identify effective cancer treatments [14]. Moreover, the study by Sampaziotis, F. et al. [15]on the repair of bile ducts in the human liver after bile duct cell organ transplantation also confirms the value of organoids for human regenerative medicine. In recent years, organoids technology has been growing in use in various fields, and the number of publications about it is increasing yearly Although organs have shown excellent potential in preclinical studies, however, there are several challenges that must be addressed in this area, such as costly and time-consuming and low success rates. Thus, there is a need for bibliometric analysis of organoid research to summarize and analyze the existing research base, identify research hotspots, and overcome current clinical deficiencies.

In academic research, research performance is often rated by chapter bibliometric indicators (number of publications and number of citations). The bibliometric approach is widely used because it is compact, easy to handle and objective in its information [16]. Coupling the use of modern computer technology to visualize the data, is more helpful in uncovering the intrinsic links between information. Therefore, it is possible to assess research activity and trends on specific topics and the most prominent research trends in future research using bibliometric analysis, which is not limited to the literature itself but also to authors, keywords, institutions, countries, etc. [17]. The class organ has grown by leaps and bounds in the last 20 years, but there is a scarcity of articles in this area of bibliometric analysis. Therefore, the aim of this study is to analyze the overall situation of organ-related research through three bibliometric software, VOS viewer, R2 Advanced and CiteSpace, to identify research trends and frontier hotspots in the last two decades and to provide a reference for researchers to understand the corresponding fields and find collaborations.

## 2 Materials and methods

### 2.1 Data collection

WoS (Web of Science) is a high-standard digital database that is now accepted by researchers almost worldwide and has become a normative tool for searching and evaluating different types of publications [18]. It is widely cited as a dataset for large-scale data-intensive research in a diversity of fields [19]. Therefore, we selected Web of science to search and collect all types of literature concerning organoids. The search strategy syntax contained was TS= (organoid OR organoids), the search deadline was January 7, 2023, the types of literature searched included Article and Review, and the language was restricted to English for later analysis of the literature content. A total of 13,143 English publications, including 9952 articles and 3191 reviews, were all downloaded in text format from the WoSCC database. The complete record of each publication includes publication results, research category, country/institution, author/co-author, journal, references, and keywords. The exact process of searching is shown in the flow chart (Fig. 1).

**Fig. 1:**
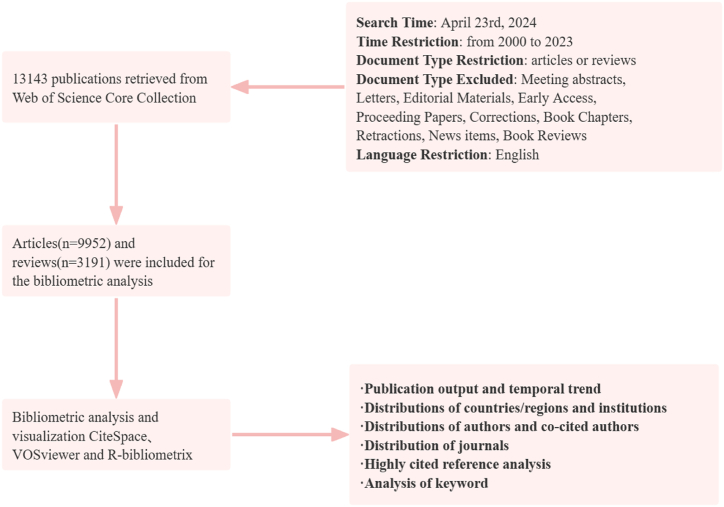
Flowchart of study retrieval and selection.

### 2.2 Data analysis

We merged keywords with the same author's name and meaning. Subsequently, we imported the processed data into CiteSpace version 6.1. R2, VOSviewer v.1.6.18.0, Bibliometrix [20] AND TBtools for bibliometric analysis. CiteSpace is based on a set-theoretic approach to data normalization to measure the similarity of knowledge units [21]. VOSviewer pays special attention to the graphical representation of bibliometric maps. It displays many bibliometric maps in an accessible way, with beautiful images and straightforward mapping [22].

## 3 Results

### 3.1 Publication output and temporal trend

Organoids is an emerging technology, so until 2010, the annual number of publications outputs was limited to around 20–30. However, from 2011 to 2021, the growth rate accelerated rapidly. However, growth accelerates rapidly from 2011 to 2023.2011, for example, there was an 84.6 % increase over the previous year, and the growth continued over the following decade. The number of annual publications peaks at 2397 b y 2021 (Fig. 2) and stabilizes at over 2600 b y 2022. At the same time, the annual citation frequency of the field is also growing.

**Fig. 2:**
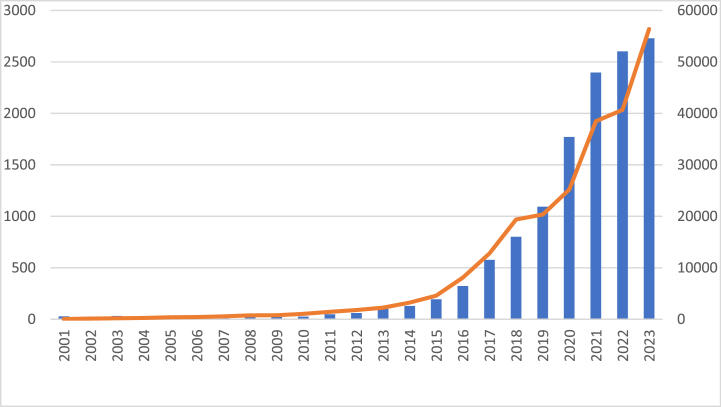
The number of organoids-related publications and citation frequency for each year from 2000 to 2023.

### 3.2 Distributions of countries/regions and institutions

To identify some countries that have made significant contributions to the field of the organoid, we analyzed the volume of publications from 86 countries or regions and 6129 research institutions. Ten of these countries published more than 4 % of the publications, all belonging to the Northern Hemisphere (Fig. 3A). The USA had the highest number of publications (4261) and citations (160,522), followed by China (1508, 15.27 %) and Germany (1090, 11.03 %). In terms of Total Link Strength, the US (2719), Germany (1325), and the UK (1126) were the strongest (Table 1). It is worth noting that the linkages between countries/regions are concentrated between countries such as the United States, Germany, China, Japan, the United Kingdom and the Netherlands. We find that the links between countries with more publications are much closer than those with fewer publications. For example, the USA, the country with the highest number, mainly co-authored with the Netherlands, followed by China and Germany, which were among the top ten countries regarding the volume of publications (Supplementary Fig. S1).

**Fig. 3:**
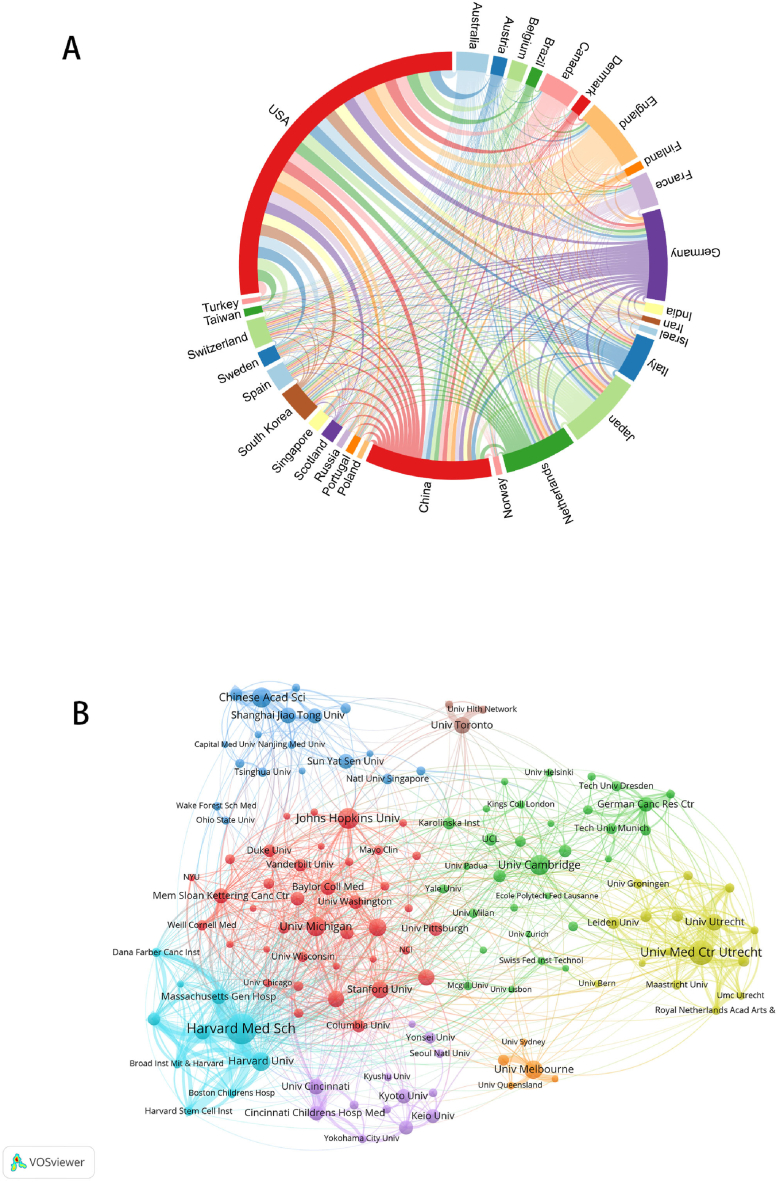
Countries/regions contributing to organoid research. (a) Links between countries with the leading number of publications. The curve between countries reflects the closeness of collaboration, with thicker lines indicating closer collaboration. (b) Nodes of different colors in the graph represent institutions in different clusters, and the size of the nodes indicates their frequency of occurrence. The thicker the line, the greater the number of collaborative publications between the two institutions.

**Table 1:** Top 10 countries/regions in terms of the number of publications, and the corresponding frequency of citations.

Rank	Countries	Documents	Countries	Total Link Strength	Countries	Citations
1	USA	4261	USA	2719	USA	160,522
2	China	1508	Germany	1325	Netherlands	59,204
3	Germany	1090	England	1126	England	34,508
4	Japan	903	Netherlands	975	Germany	29,196
5	Netherlands	860	China	922	Japan	25,575
6	England	798	Italy	702	China	25,325
7	Italy	539	Japan	572	Canada	15,146
8	Canada	429	Switzerland	569	Austria	14,326
9	France	407	France	541	Italy	14,287
10	South Korea	406	Canada	481	Switzerland	14,207

There were 4125 institutions that published documents in the field of organoids. We have produced Table 2 which clearly displays the top 10 institutions in terms of the number of publications, frequency of citations and total link strength. The institution with the greatest number of articles is Harvard Med Sch in the USA (332 publications), followed by Univ Med Ctr Utrecht in the Netherlands (241 publications). In the top ten in terms of the number of publications, seven institutions are from the United States, more than half of the total. The institution most frequently cited is Univ Med Ctr Utrecht, with 36,982 citations, far more than Harvard Univ, indicating that it is the most recognized institution. In terms of co-authorship, Harvard Med Sch had the highest total link strength at 811. In summary, the U.S. was still the most robust total research input in organoids (Table 2). We also visualized and analyzed collaborations among institutions using Citespace 6.1, R2 Advanced and VOSviewer. Notably, the institutions with the strongest collaborations are all from the same country, which could be more conducive to the exchange between countries and the rapid development of the organoids field (Fig. 3B). Supplementary Fig. S2 showed Chinese Acad Sci, Shanghai Jiao Tong Univ, and German Canc Res Ctr had only recently started research in the organoid field. In contrast, Univ Med Ctr Utrecht, Univ Michigan and Harvard Univ published a considerable number of papers and were early starters in this field.

**Table 2:** Top 10 institutions in terms of the number of articles published, the strength of association and number of citations.

Rank	Institution	Original country	Publications	Institution	Original country	Total Link Strength	Institution	citations	citations
1	Harvard Med Sch	USA	332	Harvard Med Sch	USA	811	Univ Med Ctr Utrecht	Netherlands	36,982
2	Univ Med Ctr Utrecht	Netherlands	241	Univ Med Ctr Utrecht	Netherlands	462	Harvard Univ	USA	16,282
3	Johns Hopkins Univ	USA	192	Harvard Univ	USA	397	Harvard Med Sch	USA	15,573
4	Chinese Acad Sci	China	184	Massachusetts Gen Hosp	USA	338	MIT	USA	12,437
5	Univ Cambridge	USA	182	MIT	USA	326	Univ Cambridge	England	11,504
6	Univ Michigan	USA	169	Brigham & Womens Hosp	USA	290	Johns Hopkins Univ	USA	10,880
7	Harvard Univ	USA	163	Mem Sloan Kettering Canc Ctr	USA	279	Mem Sloan Kettering Canc Ctr	USA	9477
8	Univ Melbourne	Australia	160	Univ Utrecht	Netherlands	256	Royal Netherlands Acad Arts & Sci	Netherlands	9359
9	Univ Calif San Francisco	USA	152	Chinese Acad Sci	China	253	Stanford Univ	USA	8920
10	Stanford Univ	USA	147	Univ Cambridge	England	240	Univ Michigan	USA	8293

### 3.3 Distributions of authors and co-cited authors

**Fig. 4:**
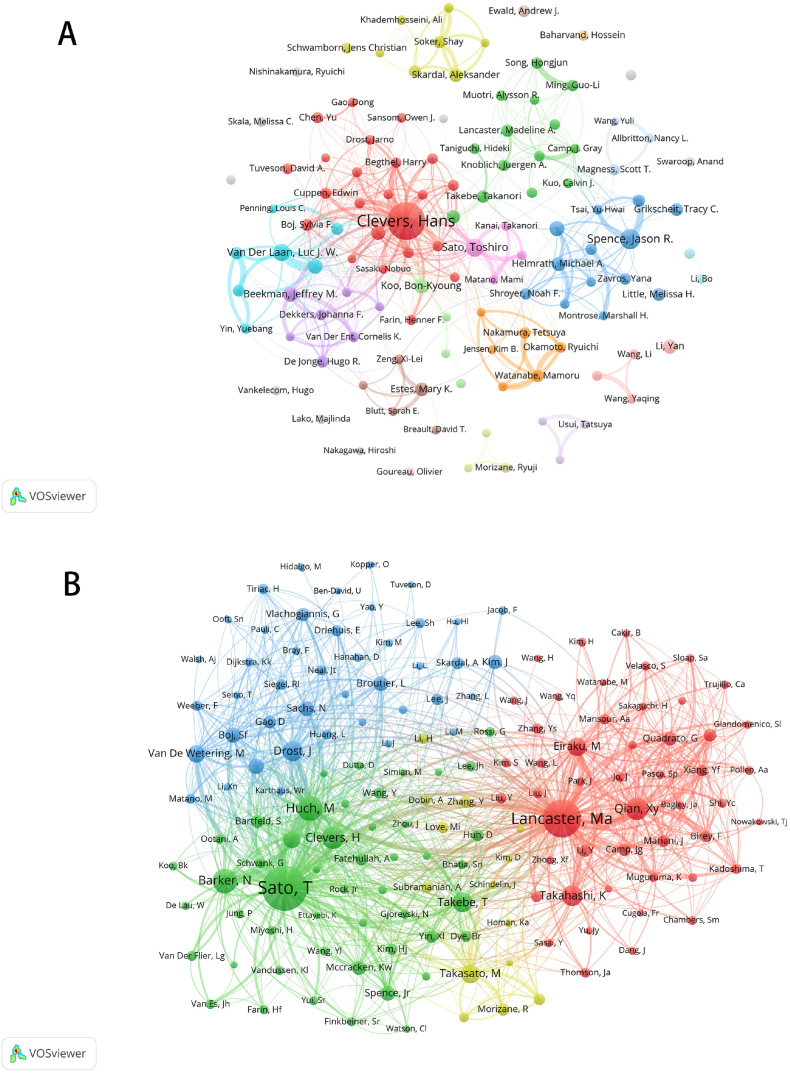
Analysis of authors related to organoids. (a) Network visualization diagram of collaboration among authors; (b) Network of co-cited authors. Sizes of nodes represent co-citations of authors, co-cited authors were defined as at least two authors who are referred to in at least one ensuing paper, and they are said to have a co-citation relationship.

An analysis of the authors of publications on organoids gives us an insight into the representative scholars and core strengths of the field. Table 3 shows a list of the top ten authors of organoid studies. In terms of volume, Clevers, Hans from the Hubrecht Institute in Finland had the largest number of distributed publications (192 articles), followed by Spence and Jason R (64 articles), and his team was the first to culture small intestine organs in vitro successfully. He was also among the top ten authors regarding the frequency of co-citations, which indicates his significant contribution and high reputation in the field. Besides he and Spence and Jason R, the number of publications published by other researchers is almost 30 to 50, with a slight difference. Sato, Toshiro from Keio University, Japan, had the largest co-citations. Overall, the collaboration between the authors was fragmented and mainly centred on Clevers, Hans. However, the top ten authors had little collaboration with each other, and the only close collaborators were Clevers, Hans and, Van Der Laan, Luc J. W. (Fig. 4A). The different colored sections of the co-cited authorship network reflect the same characteristics in co-cited authorship studies (Fig. 4B). Apart from the yellow group (Takasato, M, Morizane, R and Love, Mi), which contains fewer researchers, the three groups were well-distributed in terms of the number of research directions and were very closely related to each other.

**Table 3:** Top 10 authors in terms of number of publications and frequency of co-citations.

Rank	Author	Documents	Total link strength	Countries/regions	institution	Author	co- citations	Total link strength	Countries/regions	institution
1	Clevers, Hans	192	357	Netherlands	Hubrecht Institute	Sato, T	4191	47,477	Japan	Keio University
2	Spence, Jason R.	64	81	USA	University of Michigan	Lancaster, Ma	3166	47,926	UK	MRC Laboratory of Molecular Biology
3	Sato, Toshiro	53	123	Japan	Keio University	Huch, M	1563	27,825	German	The Max Planck Institute of Molecular Cell Biology and Genetics
4	Van Der Laan, Luc J. W.	47	113	Netherlands	Erasmus University	Barker, N	1510	18,845	UK	Sheffield Children's Hospital NHS Foundation Trust
5	Koo, Bon-Kyoung	38	56	England	University of Cambridge	Qian, Xy	1299	23,533	China	Shanghai University of Traditional Chinese Medicine
6	Beekman, Jeffrey M.	37	87	Netherlands	Utrecht University	Clevers, H	1286	17,801	Netherlands	Hubrecht Institute
7	Wells, James M.	37	48	USA	Cincinnati Children's Hospital Medical Center	Takahashi, K	1150	15,126	Japan	Kansai Medical University
8	Verstegen, Monique M. A.	35	93	Netherlands	Erasmus MC-University Medical Center	Drost, J	1100	19,671	Netherlands	Princess Máxima Center for Pediatric Oncology
9	Helmrath, Michael A.	34	75	USA	Cincinnati Children's Hospital Medical Center	Takebe, T	1075	18,735	Japan	University of Cincinnati College of Medicine
10	Huch, Meritxell	34	89	England	University of Cambridge	Takasato, M	978	15,651	Australia	The Royal Children's Hospital Melbourne

### 3.4 Distribution of journals

**Fig. 5:**
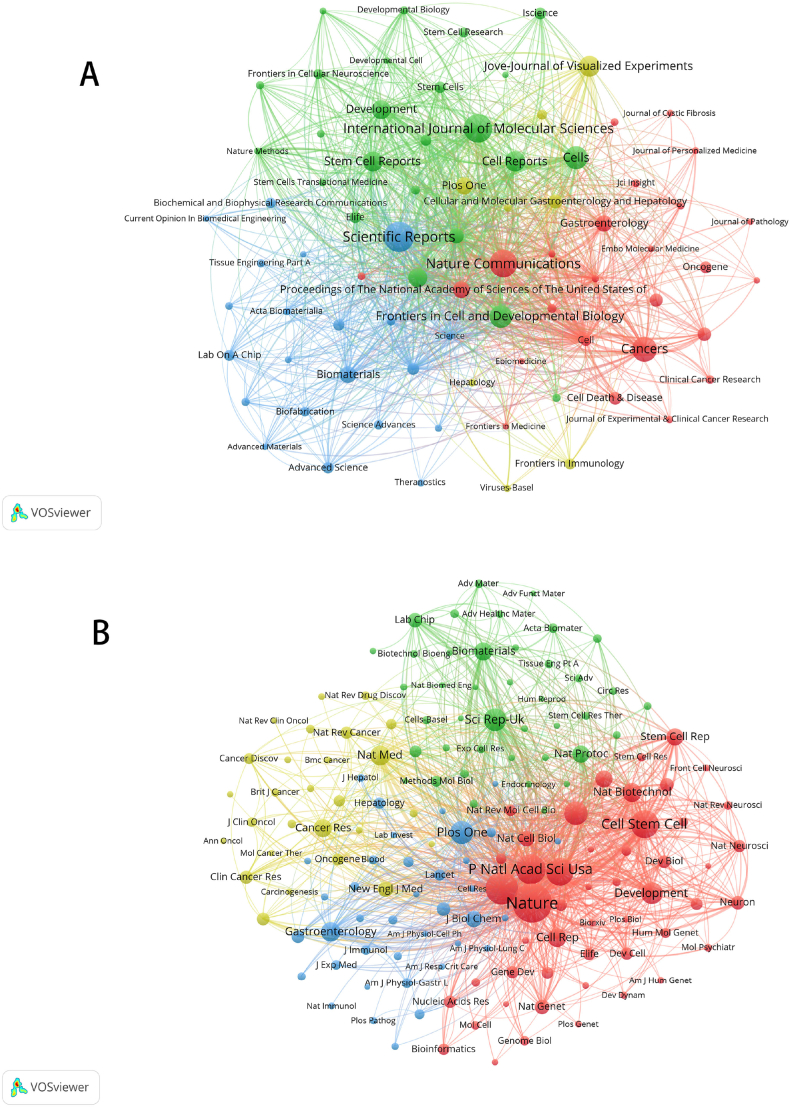
(A) Journals related to organoids; (b) co-cited journal related to organoids.

To better understand the status of publications related to organoids, we analyzed the publication status of major journals in this field. As presented in Table 4., Scientific Reports (4.997, Q2) had the most publications (n = 249), then come the International Journal of Molecular Sciences (6.208, Q1) with 224. In addition, eight of them had an IF (impact factor) of more than 5, basically in Q1 and Q2. This indicates that they all have a high academic reputation in the field. There is a positive research trend in organoids, which are more favored by journals with high impact factors. The most frequently cited journal is Nature from the USA (69.504, Q1), with a total of 32,133 citations, followed by Cell from the UK (66.85, Q1) and P Natl Acad Sci Usa from the USA (12.779, Q1), all of which have a significant position in the field of organoids. Fig. 5A visualizes the most frequently cited journals, where there is some similarity between journals of the same color. The journals in red are Nature Communications, Cancer, and Gastroenterology, which focus on the organoids in cancer. The journals in blue, Scientific Reports, Biomaterials and Biofabrication, focus on the study of the materials of 3D culture. Stem Cell Reports and Cells focus on research at the molecular and cellular levels. The yellow journals Jove-Journal of Visualized, Plos One and Frontiers in Immunology focus on the use of organoids in immunology. We can observe a strong link between them, suggesting that the various research directions in organoids are complementary and interpenetrating. Each circle in Fig. 5B represents a journal; the circle's size depends on the association's strength and the number of references. Journals with many co-citations indicated a strong association with each other. For example, Nature, P Natl Acad Sci Usa, and Cell Stem Cell have more co-citations and influence. The journals in this category all belong to the type related to organoid culture raw materials, indicating that this is the basis of research in the field of organoids.

**Table 4:** Top 10 countries/regions in terms of the number of publications, and the corresponding frequency of citations.

Rank	Journal	Publications	IF(JCR2020)	JCR quartile	Co-Cited-Journal	Citations	IF(JCR2020)	JCR quartile
1	Scientific Reports	249	4.997	Q2	Nature	32,133	69.504	Q1
2	International Journal of Molecular Sciences	224	6.208	Q1	Cell	21,795	66.85	Q1
3	Nature Communications	217	17.694	Q1	P Natl Acad Sci Usa	18,214	12.779	Q1
4	Cancers	184	6.575	Q1	Cell Stem Cell	16,092	25.269	Q1
5	Cells	164	7.666	Q2	Science	15,685	63.832	Q1
6	Frontiers in Cell and Developmental Biology	159	6.081	Q2	Nat Commun	11,373	17.694	Q1
7	Jove-Journal of Visualized Experiments	142	1.424	Q3	Plos One	10,832	3.752	Q2
8	Cell Reports	136	9.995	Q1	Sci Rep-Uk	10,629	4.997	Q2
9	Stem Cell Reports	134	7.294	Q2	Development	9613	6.862	Q1
10	Development	126	6.862	Q1	Nat Med	8244	87.244	Q1

### 3.5 Highly cited reference analysis

**Fig. 6:**
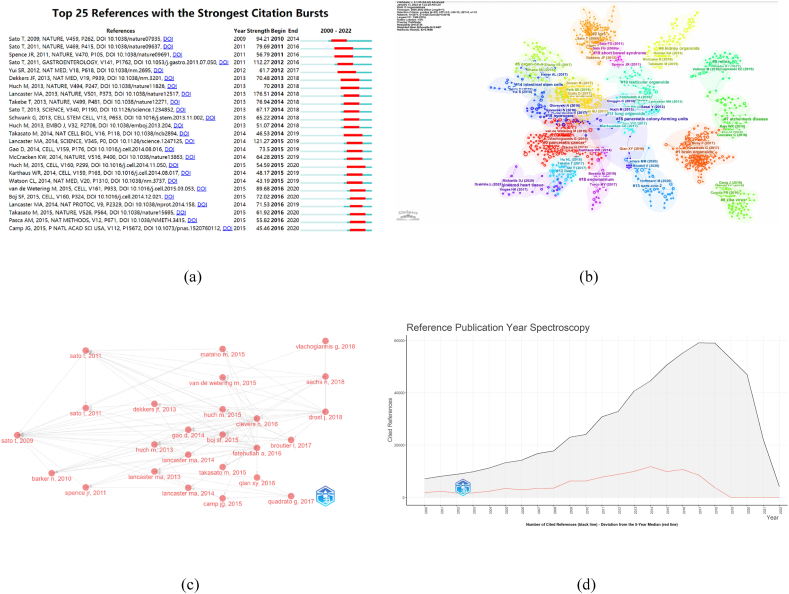
(A) CiteSpace visualization map of top 25 references with the strongest citation bursts involved in organoids; (b) cluster view of co-cited references in organoids; (c) The articles with the highest total citations are listed in chronological order from left to right. The red dots represent articles, and the grey lines between the dots represent links between articles; (d) The x-axis is the year of publication of all literature in the field of organoids, and the y-axis is the total citations of literature published in that year. (The main y-axis is the total frequency of citations; the secondary axis is the 5-year moving average of the total frequency of citations, with fluctuations in the secondary axis revealing the year of publication of key literature, i.e., literature published in that year is cited more frequently than the year before or after).

By analyzing the literature of the top 15 co-cited (Table 5) and the top 25 strongest citation bursts references on organoids (Fig. 6A), We are able to detect the dynamic changes of research directions in this field and give some indication of future trends. We used CiteSpace to categorize the co-cited references into different clusters to give a better overview of how the topic has evolved in the field and how much research is currently going on in each direction (Fig. 6B). There are 19 different clusters in total. If the number of articles belonging to this cluster is larger, its serial number is smaller. In this case, the largest cluster is the red cluster labelled #0 for pancreatic cancer, which is closely associated with #15 hydrogels and #12 liver. We can get an overview of the evolution in organoids from the year of publication of the articles represented in each cluster. The most influential publications of the last two decades can be divided into the early and the more recent parts. Early research in this field mainly focused on #2 lgr5, #19 short bowel syndrome and #16 pancreatic colony-forming units. During this period, Sato, T. published influential papers on Lgr5 stem cells, which are cells that express the LGR5 gene, one of the earliest topics. His publication “Single Lgr5 stem cells build crypt-villus structures in vitro without a mesenchymal niche', published in 2009. They stimulated the generation of crypt-villus organoids by Lgr5+ stem cells and concluded that the intestinal crypt unit as a self-organizing structure can be established by a single stem cell in the absence of a non-epithelial niche. ([23]. Not only does this publication have the most co-cited references, but it is also the strongest burst one. Huch M et al. [24] used Lgr5+liver progenitor cells to culture the three-dimensional liver bud structure. Furthermore, Yui SR et al. [25]demonstrated the feasibility of colon stem-cell therapy by using organoid technology to grow single Lgr5+ colon stem cells in vitro. These papers were cited in a 2018 review by Drost J et al., assessing the feasibility and benefits of oncologic organoid protocols and outlining ways to use organoids as cancer research models for research [26](Fig. 6C). Regarding short bowel syndrome, in 2009 Sala FG et al. [27] successfully generated tissue-engineered intestines (TESI) with stem cell niche characteristics. Two years later, they performed this technique in mouse models, laying the foundation for later human treatment [28]. Researchers studied pancreatic colony-forming units mainly in 2013. The experiments of Greggio C et al. [29]successfully constructed the culture conditions suitable for the culture of organoids, and cultured hollow spheres mainly composed of pancreatic progenitor cells in Matrige by adjusting the components of the medium. After subsequent experiments, they also found that the cultured hollow spheres differentiated spontaneously. In the same year, another research showed that single isolated ductal cells that contain clonally expandable Lgr5 stem/progenitor cells could also differentiate into pancreatic organoids [30]. In contrast, Sasai Y's review details critical advances in developing endothelial tissue in culture [31]. Combined with the three small peaks in Fig. 6D curve in 2009, 2011 and 2013, we believe that the three directions mentioned have set the stage for the rapid development of research in organoids at a later stage. The most recent popular publications are related to establishing disease models using organoid technology ([27,28]. Most recent high-impact articles are related to disease modelling using organoids technology. In 2020, the two articles cited the most frequently used organoids cultured in vitro to analyze the mechanisms of SARS-CoV-2 infection of systems other than the lung. They demonstrate that SARS-CoV-2 causes damage to cardiovascular and intestinal epithelial cells by infesting entrance ACE2, providing substantial evidence for multi-organ failure in severe cases of COVID-19 [32,33]. We also found that organ-like models of the gut, brain and kidney have long been established, but only recently has a similar approach been developed for the heart. Moreover, researchers modelled hypoxia-enhanced adriamycin cardiotoxicity through heart-forming organoids (HFOs), which could aid drug screening and development ([34,35]. We noticed that recent publications with strong bursts had been related to the brain's nervous system. The publication " Cerebral organoids model human brain development and microcephaly' by Lancaster, MA, is one of the most cited in this direction, with 2505 citations. In their experiment, to overcome the difficulty of replicating microcephaly in mice, they tried to develop a three-dimensional organoid culture system derived from human pluripotent stem cells and finally succeeded in simulating microcephaly [36]. Furthermore, two publications with the most recent burst proved that the cell types of brain-like organoids are well represented in the developing human cortex and exhibit specific physiological properties of neural cells, such as neuronal activity [37,38]. The use of organoids in the brain and nervous system is expected to be a popular direction for future research.

**Table 5:** Top 15 co-cited references related to organoids.

Rank	Author	Article Title	Source Title	Cited	Year	Category	DOI
1	Sato, T	Single Lgr5 stem cells build crypt-villus structures in vitro without a mesenchymal niche	NATURE	3847	2009	Article	10.1038/nature 07935
2	Lancaster, MA	Cerebral organoids model human brain development and microcephaly	NATURE	2505	2013	Article	10.1038/nature 12517
3	Sato, T	Long-term Expansion of Epithelial Organoids from Human Colon, Adenoma, Adenocarcinoma, and Barrett's Epithelium	GASTROENTEROLOGY	1963	2011	Article	10.1053/j.gastro.2011.07.050
4	Sato, T	Paneth cells constitute the niche for Lgr5 stem cells in intestinal crypts	NATURE	1623	2011	Article	10.1038/nature 09637
5	Lancaster, MA	Organogenesis in a dish: Modelling development and disease using organoid technologies	SCIENCE	1375	2014	Review	10.1126/science.1,247,125
6	Clevers, H	Modelling Development and Disease with Organoids	CELL	1322	2016	Review	10.1016/j.cell.2016.05.082
7	Monteil, V	Inhibition of SARS-CoV-2 Infections in Engineered Human Tissues Using Clinical-Grade Soluble Human ACE2	CELL	1281	2020	Article	10.1016/j.cell.2020.04.004
8	van de Wetering, M	Prospective Derivation of a Living Organoid Biobank of Colorectal Cancer Patients	CELL	1241	2015	Article	10.1016/j.cell.2015.03.053
9	Spence, JR	Directed differentiation of human pluripotent stem cells into intestinal tissue in vitro	NATURE	1159	2011	Article	10.1038/nature 09691
10	Qian, XY	Brain-Region-Specific Organoids Using Mini-bioreactors for Modelling ZIKV Exposure	CELL	1128	2016	Article	10.1016/j.cell.2016.04.032
11	Boj, SF	Organoid Models of Human and Mouse Ductal Pancreatic Cancer	CELL	1118	2015	Article	10.1016/j.cell.2014.12.021
12	Barker, N	Lgr5 (+ve) Stem Cells Drive Self-Renewal in the Stomach and Build Long-Lived Gastric Units In Vitro	CELL STEM CELL	1043	2010	Article	10.1016/j.stem.2009.11.013
13	Ohlund, D	Distinct populations of inflammatory fibroblasts and myofibroblasts in pancreatic cancer	JOURNAL OF EXPERIMENTAL MEDICINE	1003	2017	Article	10.1084/jem.20,162,024
14	Huch, M	In vitro expansion of single Lgr5 (+) liver stem cells induced by Wnt-driven regeneration	NATURE	918	2013	Article	10.1038/nature 11826
15	Lamers, MM	SARS-CoV-2 productively infects human gut enterocytes	SCIENCE	894	2020	Article	10.1126/science.abc 1669

### 3.6 Keyword analysis

The analysis of keywords in bibliometrics is one of the critical indicators to find the frontier of the research field. Table 6 shows the top 20 keywords with the highest occurrence times. All these keywords are related to each other in some way. Culture organoids needs adult stem cells (ASCs) and Pluripotent stem cells (PSCs), such as Embryonic stem cells (ESCs), Induced pluripotent stem cells (IPCs) and induced pluripotent stem cells (IPCs) [39]. At the same time, specific small molecules and growth factors, such as extracellular matrix, fibroblast growth factor (FGF), Wnt, Noggin, etc., are also essential [[40], [41], [42]], and through techniques such as 3 d cultures, tissue engineering and microfluidics, cultures can brain organoids, intestinal organoids, patient-derived organoids and cerebral organoids [43], which can be used in disease models, drug screening, colorectal cancer, personal colorectal cancer, personalized medicine, cancer and precision medicine [44,45].

**Table 6:** Top 20 keywords with the highest occurrence times.

Rank	Keyword	Occurrences	Total link strength	Rank	Keyword	Occurrences	Total link strength
1	organoid	2072	3323	11	ips cells	161	318
2	stem cells	446	919	12	spheroids	158	357
3	3 d cultures	327	639	13	intestinal organoids	157	147
4	tissue engineering	249	494	14	cancer	150	310
5	induced pluripotent	243	438	15	personalized medicine	150	366
	stem cells						
6	disease models	218	534	16	differentiation	142	276
7	colorectal cancer	215	269	17	in vitro models	141	271
8	brain organoids	193	311	18	human pluripotent stem cells	134	195
9	drug screening	178	402	19	organ-on-chip	134	330
10	pluripotent stem cells	170	316	20	microfluidics	133	311

**Fig. 7:**
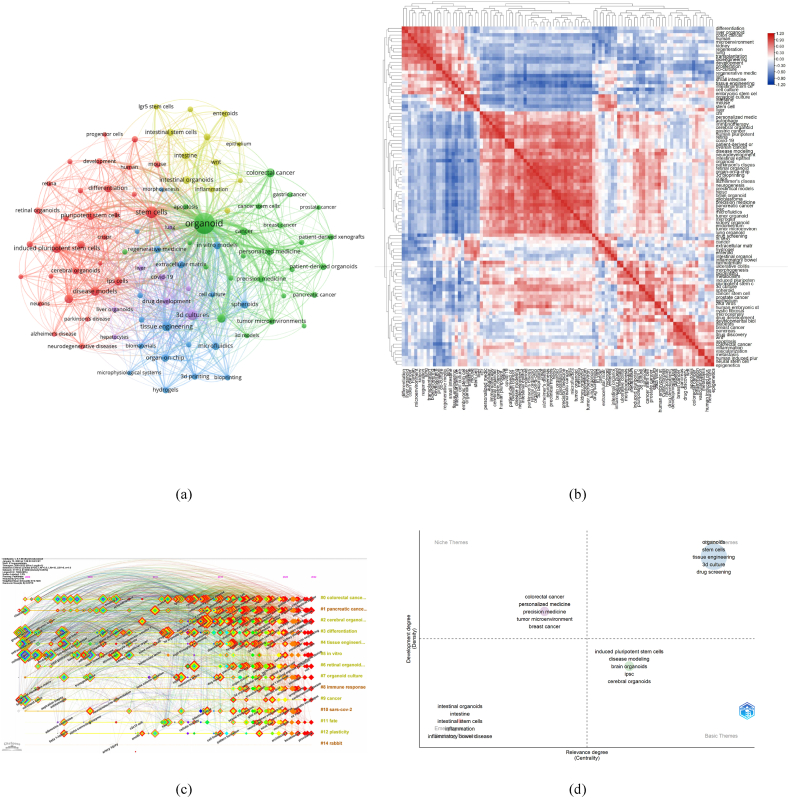
(A) Network clustering map of keywords' co-occurrence analysis based on VOSviewer. The minimum threshold for the number of keyword frequencies is 40. Keywords were divided into 5 clusters: cluster 1 (red), cluster 2 (green), cluster 3 (blue), cluster 4 (yellow), and cluster 5 (purple). Each keyword is represented as a node, and the node size is proportional to the frequency. The line between nodes denotes a co-occurrence relationship. The distance between nodes denotes the degree of relevancy, and the closer the distance, the higher the degree of relevancy. (b) Heat maps showing the co-occurrence of keywords. (c) A timeline view of the keyword analysis from 1991 to 2022 plotted by CiteSpace. The elements on the horizontal axis represent keywords; the position of the element on the horizontal axis indicates the time of first occurrence; the line connecting the elements indicates the relationship of the keywords. The size of the elements is proportional to the number of references cited. (d) Thematic map of related research. Words in the upper right corner (first quadrant) are motor themes. In the upper left corner (second quadrant) are niche themes, in the lower left (third quadrant) are emerging or declining themes, and in the lower right (fourth quadrant) are basic themes.

To further visualize the links between keywords, we have grouped them using VOSviewer (Fig. 7A), with five clusters in total and different colors representing different clusters. It is clear that the links between cerebral organoids, disease models, ips cells and induced pluripotent stem cells in the red cluster are solid, as are the links between 3 d cultures, drug development, tissue engineering and organ-on-chip in the blue and purple clusters. Supplementary Fig. S4 illustrates the links between keywords and the chronological order in which they were used, with the bluer the color indicated the earlier the study, and the redder the color indicating that the keyword belongs to a more recent research direction. The research hotspots in the field of organoids have changed dramatically. In recent years kidney organoids, patient-derived organoids, ovarian cancer, precision medicine, immunotherapy, and the tumor microenvironment have all represented emerging frontiers, most of which have only been gaining attention in the last two years. In contrast, organoid culture, morphogenesis, neural stem cell and lgr5 have been studied less recently, but most have been studied earlier, in some cases as early as 2005. Current research has gradually shifted from the materials and techniques required to cultivate organoids (organoid culture, morphogenesis, neural stem cell and lgr5) to the applications of organoids (kidney organoid, patient-derived organoid, ovarian cancer, and precision medicine). Fig. 7B and Supplementary Fig. S3 show the correlations between the more popular keywords from 2000 to 2022. Among the main ones are the construction of organoids and the culture of organoids with raw materials correlated very closely. For example, neurodevelopment is closely related to human embryonic stem cells, iPSC and pluripotent. It indicated that brain organoids in neurodevelopment are mainly derived from human embryonic stem Disease modelling and bioprinting are often associated with articles on cystic fibrosis and pluripotent stem cell. Drug discovery often appears alongside keywords such as breast and colorectal cancer, indicating that organoids can be used in drug development for various cancers. The keywords organ-on-a-chip and 3 d bioprinting frequently appear in articles on spheroids and 3 d culture. The network clustering map (Fig. 7C) shows the evolution process of each cluster by marking the first occurrence time of each keyword. The most frequently used cluster is currently #0 colorectal cancer, and #5 in vitro belongs to the earliest direction studied. Notably, #10 sars-cov-2 and #14 rabbit are no longer studied. Fig. 7D visualizes four different types of themes according to two dimensions (density and centrality). The Motor Themes in the upper right quadrant represent directions that are important and already rapidly developing in the field of organoids, which is the focus of current research. The Basic Themes in the lower right quadrant represent topics that are important to the field of organoids but are not yet well developed and will be the focus of future research in the field of organoids.

### 3.7 Factorial analysis

**Fig. 8:**
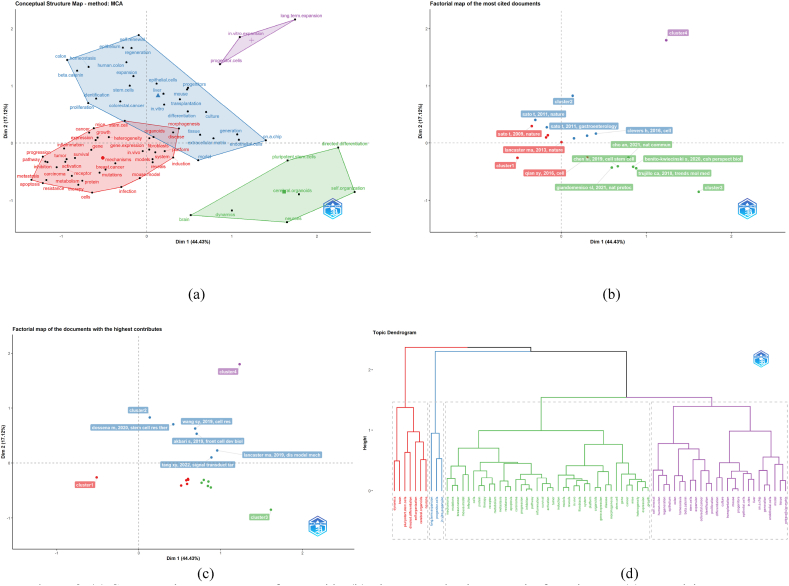
(A) Conceptual structure map of organoids. (b) The connection between the four clusters. (c) Factorial map of the most cited documents. (d) Factorial map of the documents with the highest contributions.

General citation analysis can lead to the loss of articles with prominent contributions and high citation rates due to different topics [46]. Therefore, we used factorial analysis, which will more comprehensively analyze the publications related to the critical themes. We divided the research areas into four categories and showed the links between the keywords in these four categories (Fig. 8A and. B). The words in the blue clusters relate to the process of organoids in culture. Most models of cultured organoids are derived from progenitor cells and are obtained by long-term expansion in vitro. The red clusters represent words related to organoid species. Most of them, like the zika virus infection, identification of the developmental roots of autism and the formation of the neurological barrier in the brain requires cerebral organoids [47,48]. Purple clusters identify the characteristics of organoids. It can build and grow from a single cell for months while retaining key morphological, functional and gene expression features [49]. Green clusters include organoids for a variety of applications. Fig. 8C is obtained by analyzing Bibliometrix, which presents the factorial map of the most cited articles in each cluster. As the figure shows, the documents are divided into 3 clusters across 2 dimensions or factors. Cluster 3 represents the most cited articles published recently. By analyzing the articles by Silvia Benito-Kwiecinski et al. [50], Truillo ca. et al. [51], Chen hi et al. [52], and Giandomenico SL et al. [53], it can be determined that recent research in the field of organoids has had breakthroughs in the direction of Brain Organoids and this will be the future of Hotspots and frontiers in this field. The six publications in Fig. 8D are all in the positive quadrant of both dimensions, so they all have a high contribution level. The highest contributions were made by Dossena M et al. [54] in 2020, which described a technique for cultivating the Isolation of human pancreas organoids (hPOs) from discarded pancreatic tissue.

## 4 Discussion

This study conducted visual research on the field of organoids, and presented the current research status and hotspots in this field. We used CiteSpace 6.1. R2 Advanced, VOSviewer 1.6.18, and *R*-bibliometrix to analyze data from 9884 articles on organoids in the web of science and to assess the spatial and temporal distribution of the field, author contributions, core articles, research hotspots and frontiers.

### 4.1 General distribution

According to qualitative and quantitative analyses by CiteSpace and VOSviewer, organoid publications have continued to grow, with a minor increase between the beginning of the 21st century, possibly related to the development of stem cell research, as organoids are cultured from PSCs and iPSCs [55,56]. However, since 2009, when Sato et al. built the first human tissue-derived 3D intestinal organ, organoids have received a great deal of attention [57]. After that, the number of relevant articles in the field of organoids grew almost exponentially and peaked in 2021.

The United States is a decisive player in the field of organoids research, ranking first in terms of the number of publications, citation frequency and Total Link Strength. In contrast, although China has many publications, ranking second, both the frequency of citations and Total Link Strength are unsatisfactory, indicating that China needs to improve its research quality and reference value. In addition, the rest of the top 10 countries, except for China, are all developed countries, which is closely related to the investment in research in each country, probably due to differences in socio-economic status, general research capacity and population size [58] It is also significant for cooperation between countries, which can reflect the degree of co creation between regions [59]. There is close cooperation between these six countries: the USA, China, Japan, Germany, Netherlands, and the UK and we also note that these six countries are among the top six in terms of the number of articles published as well as the frequency of citations. In terms of institutions, 60 % of the top 10 cited institutions are in the USA, followed by the Netherlands (n = 2, 20 %), while the top three institutions have been working on organoids for a long time and are less active in the field recently. We have also found good collaborations between some institutions, such as Univ Med Ctr Utrecht, Univ Utrecht and Massachusetts Gen Hosp. These closely linked institutions are almost all from the same country, with little collaboration between institutions in different countries, which in the long term will hinder the development of the research in the long run, and this situation will hinder the development of the research field. Thus, we strongly recommend extensive collaboration and communication between national research institutions to develop organoids.

Clevers, H is one of the authors who has made an outstanding contribution to the field of organoids, with the highest number of publications and far more than anyone else. Sato, T has also contributed significantly to the field, being the most co-cited author and having three articles ranked first, third and fourth, respectively. He successfully produced mouse intestinal-like organs in 2009. By analyzing past research results on their growth and maintenance needs, they identified several elements crucial for stem cell maintenance, namely Wnt, EGF, Noggin and *R*-spondin1 (Rspo) [23]. Wnt proteins, a class of secreted glycoproteins widely found in animals, are critical for stem cell self-renewal and influence development and tissue regeneration. EGF, or Epidermal Growth Factor, promotes cell proliferation and differentiation and maintains the active state of stem cells. Noggin inhibits BMP signaling, which helps to keep stem cells from differentiating. In addition, *R*-spondin1 (Rspo1), which enhances Wnt signaling, is particularly important for stem cell proliferation. In the following five years, he established human tissue-derived colon carcinoids and screened various factors by adjusting culture conditions to find elements that contribute to organoid growth and optimal culture scheme [[60], [61], [62]].

We found that most of the top ten journals in terms of the number of articles are related to molecules, cytology, and biology, such as Cell Reports, Cells, and Nature Communications. This suggests that most organoid research is still at the primary research stage and has yet to be applied significantly in the clinical setting. In addition, an analysis of the top co-cited journals can help researchers to find the core journals in the field, where articles can be used as authoritative references [63]. However, eight of the top ten journals in organoids have an IF above 5, indicating the importance of research in the field of organoids.

### 4.2 Hotspots and frontiers

The analysis of keywords helps us to identify the current hot research in the field of organoids. These keywords can be divided into four main categories: raw materials for cultivating organoids, techniques used to cultivate organoids, applications of organoids and types of organoids produced. According to the Overlay map of keywords, the latter two are the most popular areas of recent research: applications of organoids (drug screening, personalized medicine, precision medicine) and types of cultured organoids (brain organoids, patient-derived organoids, chemotherapeutic patient-derived xenografts). These themes are likely essential research directions for the future.

#### 4.2.1 Current application hotspots for organoids

##### 4.2.1.1 Cancer

The application of Organoids is now a significant area of research, and their use in cancer is receiving much attention. This technology facilitates the testing of drugs for various cancers and guides the personalized treatment of cancer. Building in vitro cancer models through tumorigenesis helps researchers observe the entire cancer infection and mutation [64,65]. Several infectious pathogenic infections are important causes of cancer, such as HPV which causes cervical cancer, HBV and HCV which cause liver cancer, RXMV which causes prostate cancer, and EBV which causes nasopharyngeal cancer [[66], [67], [68]]. However, organoids technology can provide an in vitro model demonstrating the relationship between these infectious agents and cancer. In addition, organoid technology allows the identification of mutational loci in cancers, most of which arise from cumulative mutations in disease-causing genes [69]. Hence, it is crucial to understand the mutational process of genes in tumorigenesis [70]. Ping Tan et al. [71]demonstrated that inhibition of the SIRT1-HIF axis significantly slowed the growth of bladder cancer in mice and humans through organoid technology and identifying SRT1720 as a new therapy for bladder cancer. More importantly, cultured organoids can be extended and cryopreserved for a long period, providing specimens for future studies as living tumor organoids Biobank [72]. In addition, organoids are used to evaluate the specific response of cancer patients as excellent models [73]. So far, in vitro models of various cancers have been constructed using this technology, including gastrointestinal cancer [74], colon cancer [75,76], hepatic cancer [77], bladder cancer [78], pancreatic cancer [79], prostate cancer [80] and breast cancer [81]. At the same time, it has also shown that using organoids directly as tumor models allows for the identification of the most sensitive drugs for tumors and the finding of the most suitable treatments for different individuals. Marc van de Wetering et al. [82] found that different cultures were differentially sensitive to Wnt inhibitors by culturing organoids from 20 colorectal cancer (CRC) patient tumors, suggesting that organoid technology can help design personalized treatments for cancer. Currently, most of the well-established organoid techniques mainly construct epithelial-derived tumor models. However, there are still some cancers for which this is not the case, and further studies on non-epithelial organ cultures are needed, such as primary glioblastoma [83] and childhood renal cancer [84]. The normal functioning and response fidelity of organoid systems depend heavily on accurately reproducing the complex inter-cellular interactions and extracellular matrix components in vivo in the microenvironment of their growth. Current technological tools are still deficient in this regard and cannot adequately model the delicate complexity of the in vivo environment. Taking a specific type of glioblastoma (GBM) as an example, the efficiency of generating and maintaining its corresponding patient-derived GBM-like organs (GBOs) is significantly lower compared to IDH1 wild-type tumors, revealing that the existing culture conditions and methods in these cases may need to be further fine-tuned and optimized to enhance the reliability and applicability of the organoid models [84]. In addition, in terms of technological innovation and improvement, Zhang, ZC et al. applied the research results of T-cell releaser (TEX) heterogeneity to organoid models, which is conducive to accurately simulating the impact of TEX on the tumor immune microenvironment and help optimize immunotherapy strategies and personalized drug screening [85].

##### 4.2.1.2 Drug discovery and personalized medicine

Researchers have begun studying the suitability of organoids for high-throughput drug screening procedures [86]. Typically, this screening is designed to simulate clinically applied treatments and is particularly suitable for rare diseases for which large-scale clinical trials are not possible [87]. Organoid technology allows these Patient-derived organoids' predictive drug response potential to be assessed by comparing patient responses to matched Patient-derived organoid responses. Researchers have made many efforts to verify the feasibility to realize the application of organoids in drug screening. For example, Katrin P. Guillen et al. [88] used drug screening with matched PDX and PDX-derived organoids (PDxO), which had almost identical effects on the model as the patient's response, indicating the high potential of organoids to predict drug response. Organoids can also be used for drug metabolism. Diane Ramsden et al. [89] have developed a new coculture hepatocyte model, the HepatoPac, which maintains a high level of liver function and provides insight into the metabolic processes and metabolites of drugs in the liver. Furthermore, drug toxicity testing with organoids has the advantage of being fast acting and reproducible compared to in vivo testing. It can save time and costs to quickly identify potential risk factors. Recently, the toxicity of short and long chain PFASs to the liver has been analyzed by liver organoids, and the results show that short-chain PFASs affect the morphology of organoids, leading to a reduction in cytoarchitectural complexity and abnormal cytological features, which demonstrates the sensitivity of organoids testing [90]. Organoids can identify new drugs and reveal which patients can benefit from treating certain (existing) drugs. For example, using organoids to validate genome-driven targeted therapies can be beneficial for selecting individual drugs for individual patients [91]. The study by Gilles et al. [92] demonstrated the therapeutic potential of the nanodrug TPN-21 can reduce tumor cell growth and survival in individual patient cells. They obtained these conclusions by performing microRNA (miRNA) analysis on PDX samples to determine the miRNA deregulation status of individual pancreatic ductal adenocarcinoma (PDAC) patients. Organoids can also be used to explore prospective combination therapies. Compared to monotherapy, JNJ-42756493 (FGFR inhibitor) plus AZD8055 or siolimus (mTOR inhibitor) has a more significant anti-tumor effect in organoids with FGFR3 mutations and nonsense TSC mutations [93]. Although the use of organoids in drug screening is now widespread, it still has limitations. First, although organoids can mimic some of the drug absorption, distribution, metabolism, and excretion processes, in vitro models may not fully reproduce the complex drug transport mechanisms in vivo, such as blood-brain barrier penetration and differences in the expression of metabolic enzymes. This may lead to deviations between organoid predictions of drug responses and the in vivo reality. In addition, organoid systems often cannot ideally mimic the gene networks and associations within the organ and exhibit immature or “fetus-like' features, which may limit their ability to accurately simulate specific complex disease phenotypes, especially late-onset diseases, Long-term culture and maintenance of organoids is complex, especially in the absence of suitable microenvironmental conditions, and may lead to tissue degradation and loss of function. In addition, the heterogeneity of organoids may also complicate data interpretation and affect the reliability of the final screening results of drugs [94]. Although current organoid models have limitations such as developmental immaturity and heterogeneity, the development of experimental tools and techniques is leading to the routine application of organoids in drug discovery. For example, Elaine T. Lim et al. developed Orgo-Seq, a framework for integrating bulk and single-cell RNA sequencing data from sampled brain organoids and postmortem brain tissue, which can be used to identify cell-specific driver genes associated with neurodevelopmental disorders. By identifying cell-specific driver genes associated with neurodevelopmental disorders, Orgo-Seq enhances the ability of organoid-based disease models to reproduce specific pathological processes, ensuring that the models more accurately mimic actual disease states and improving the predictive accuracy of drug screening and efficacy assessment [95].

##### 4.2.1.3 Model genetic disease and gene repairing

Organoids can be used not only in cancer but also in some genetic diseases. As early as 2013, Menendez et al. [96] used human pluripotent cells to grow multipotent neural crest stem cells (NCSCs) that mimic human diseases associated with neural crest development. More recently, organoid modelling techniques have been used extensively in related genetic diseases; for example, Tran, T. et al. [97] differentiated human pluripotent stem cells into 3D structural organoids that were transplanted into mice with even the most important filtration function of the kidney, which could be useful for further research into therapeutic candidates for the hereditary cystic kidney. In contrast, gene repair treats cancers associated with genetic mutations or cardiovascular disease and diabetes due to genetic predisposition, as well as acquired immunodeficiency syndrome (AIDS) caused by acquired viral infections, by altering the target gene [98]. With transgenic Lgr5+ stem cell-derived organs having been successfully generated and transplanted into damaged tissue, the promise of genetic repair is growing [24,25]. Recently, genome engineering such as CRISPR/Cas9 has been rapidly developed and applied to organoids. The combination of this technology with organoids could create new conditions for the study of organ development and the treatment of congenital diseases [99]. One of its advantages is the ability to facilitate the modelling of human cell-type diseases, like Mari Nakamura et al. [100], who generated iPSC from patients and used CRISPR/Cas9 to insert MAPT genes to form frontotemporal family dementia (FTD) model and found that mutant tau proteins exhibited abnormalities such as reduced levels of phosphorylation. Other studies have used CRISPR-Cas9 gene editing to induce human-derived APOL1G0/G0 and APOL1G2/G2 kidney-like organoids, and post-treatment assays have found that attenuating the cytotoxic effects of APOL1 risk variants can be achieved by inhibiting DGAT2 [101]. To gain a clear understanding of the pathological progression of the disease, researchers can use CRISPR/Cas9 for gene rescue [102]. Schwank et al. [103] were the first to use CRISPR/Cas9 technology to repair mutated genes at the CFTR locus in organoids cultured from CF patient stem cells. Other recent studies have obtained F8c-HA hiPSCs with prominent levels of FVIII activity by CRISPR/Cas9 editing and cultured liver organoids with 3D structures and demonstrated their therapeutic efficacy in animals with haemophilia A [104]. These studies all suggest that CRISPR/Cas9 can repair genetic mutations in PDO and facilitate the advancement of organoid technology to the clinical stage. However, the organoids' possible genetic and phenotypic heterogeneity, mainly when derived from patient samples, may lead to inconsistent gene editing effects across sites or cell populations. Even when organoids are successfully edited, maintaining the long-term stability of the edited state is a challenge. Over time, organoids may undergo phenomena such as cellular aging or gene repair that affect the continued expression or function of the edited genes. Notably, Ungricht R et al. performed the first genome-wide CRISPR screen in human-induced pluripotent stem cell (iPSC)-derived kidney organoids. This pioneering application combines high-throughput gene editing technology with complex three-dimensional tissue models, providing an unprecedented platform for exploring kidney development and disease-related gene function [105].

#### 4.2.2 Future application hotspots for organoids

##### 4.2.2.1 Chip-on-organoid (OOC)

Multi-Organ micro-physiological Systems (MPS) have significantly advanced in the last decade. It is often referred to as ‘Chip-on-organoid' or ‘Organoids-on-a-chip'. Compared to traditional biomimetic organ technology, it allows more precise control of the local environment and mimics the functional units of human organs in vitro, making it a more promising application [106]. From early prototypes of organoids on a chip [107], single-organ chips such as the lung [108], intestine [109]and liver [110], to multi-organ chips [111,112] that link multiple organs in tandem, researchers have gradually developed more complete and complex models with higher levels of simulation. These models have great potential for disease research, drug testing and virology applications. Furthermore, it can cascade multiple organs, which makes up for the inability of organoids to model multi-organ pathology when used for drug screening. A recent study assessed the role of raltegravir in SARS-CoV-2 using organ-on-chip. It showed the effects of this drug on organs such as the gastrointestinal, brain and lung [113]. Microfluidic chips can integrate tens of thousands of cell culture and assay units into a few square centimeters, while Chip-on-organoid microfluidic culture devices can also outline organs and their basic functions [114]. Compared to organoids, Chip-on-organoid can better mimic the immune response of hosts in the microenvironment when invaded by pathogens, A.M. Or-tega-Prieto et al. [115] took advantage of this to use 3D microfluidic liver cultures to explore the physiological processes of hepatitis B virus infection. An additional unique advantage of microfluidic organoids can establish oxygen gradients at the tissue-tissue interface so that gut-on-a-chip can be used to rapidly understand the interactions between the microbiota and the host human tissue [116]. In addition, Chip-on-organoid can be vascularized, which provides an excellent platform for some vascular studies on organs such as the lung [117], kidney [118] and brain [119]. However, the main challenges of existing OOC models are the translation of chips from lab concepts to screening platforms, the balance between complexity and practicality, etc.

##### 4.2.2.2 Vascularization of organoids

A current challenge with organoid technology is the insufficient diffusion of nutrients and difficulties in waste removal during culture, resulting in limited maturity levels and function. In contrast, the vascularization of organoids can further extend their lifespan by distributing nu2trients through capillaries, as occurs in vivo [120]. Methods for organoid vascularization can be divided into two categories, one using a 3D bio-printing strategy and printing ECs along with surrounding tissues to form external vascularization, primarily for cardiac tissues [121]and brain [122]. And the other transplanting organoids into hosts to achieve in vivo vascularization of organoids. Also, microfluidic techniques are feasible for tissue angiogenesis [123]. For example, extensive infiltration of host vessels was observed after the implantation of human brain-like organs into adult mouse brains [124]. Nowadays, patients mostly use immunosuppressants to reduce immune rejection between the donor organ and themselves. However, the patient's defiance system is also compromised in this way. If this model is used in place of organ or cell transplantation, on the one hand, functional organs can be rebuilt, and on the other hand, immune rejection can be avoided [125,126]. Also, the influence of some viruses' infected blood flow on the organism's distribution cannot make negligible. Therefore, to obtain a realistic picture of the infection process of the organism with viruses, the vascularization of organoids is an essential factor. Angiogenesis in organoids tumor models has also received much attention recently because tumor vasculature inhibits the efficacy of anticancer drugs by limiting their coverage in the tumor, common organoids tumor models do not fully restore the true in vivo response of tumors to drugs [124], and therefore Vascularization of Organoids is vital for drug screening. However, the vascular network in real organs has a highly complex and fine-grained three-dimensional structure, including branches, loops, and capillary beds. It is often tricky for organ-like techniques to accurately reproduce this complex vascular morphology and spatial layout. In addition, in the natural vascular system, blood flow is critical for maintaining vascular homeostasis, regulating vascular wall cell function, and transporting nutrients. Organoid models cannot simulate the natural hemodynamic environment, which has implications for assessing drug delivery, oxygen exchange, and other functions.

##### 4.2.2.3 Modelling of tumor–immune interactions

In addition to genetic changes in tumor cells, the microenvironment, which is made up of many non-epithelial cell types, immune cells, and stromal cells, plays a role in the growth and eventual dissemination of tumors [127]. Moreover, formerly thought-to-be incurable tumors may now be treated thanks to cancer immunotherapies including immune checkpoint blockade (ICB) and adoptive cell therapy (ACT) [128]. Hence, it is significant to establish the organoids' microenvironment to better mimic the immune system's function in tumors. The immunosuppressive role of myeloid-derived suppressor cells (MDSC) has been studied by co-culturing gastric cancer organoids and immune cells, and the results suggest that PD-L1 expression is regulated by the gastric cancer mTOR signaling pathway [129]. By modelling 3D co-culture with pancreatic cancer cells, CAF, and monocytes, the effect of tumor cells and fibroblasts on monocytes and their immunosuppressive phenotype was also investigated [130]. This method of adding immune cells to organoids is currently the most used by researchers [131]. In 2016, Boussommier-Calleja et al. [132] suggested that a microfluidic system could simulate the complex tumor microenvironment to study tumor-immune interactions. Using flow cytometry, the researchers first examined the cellular makeup of original tumors and microtumor microarrays. They found that the cellular design on the microtumor microarrays was representative of the primary tumor, followed by assays of programmed death-1 (PD-1) viability of tumor cells after treatment with inhibitors. The results showed that the cells could maintain high viability for the first 24 h, with a slight decrease at 36 h. *Anti*-PD1 control tests were carried out on tumor cells injected onto microtumor chips as opposed to tumor cells in 2D cultured 96-well plates in order to see if the 3D microenvironment may more accurately represent the drug response of the tumor. The results showed that tumor cells cultured in micro tumor chips significantly responded against PD1 [133]. This indicates that employing organoids-on-a-chip to examine tumor responses to cancer immunotherapy is technically feasible. In addition, the iCoC model could offer a fresh perspective on immunotherapy study design, providing access to the development of patient-specific drugs and ultimately improved patient outcomes.

##### 4.2.2.4 Transplantation therapy and regenerative medicine

For those with organ failure, organ transplantation is an essential kind of therapy. There are still a number of challenges, such as severe transplant rejection and a growing shortage of organ donors [134]. It has been proposed that current transplantation therapies can be improved by replacing irreversibly progressive diseased organs with homologous healthy organoids [135]. Organoid culture has recently been made possible by newly developed technologies like tissue engineering, biomaterials, microfabrication, and biofabrication [136]. Several studies have been conducted, and their feasibility has been demonstrated. Back in 2011, to treat retinal degenerative animals, Mototsugu Eiraku et al. [137] implanted 3D retinal slices made from embryonic and induced pluripotent stem cells. Then, using the same methodology, another study developed photoresponsive behavior by implanting cultured mouse iPSC-derived retinal tissue onto a model of end-stage retinal degeneration [138], which differed from previous simple cell transplantation and opened up new avenues for studying transplantation. In the same year, Rashidi et al. [139] created an organoid using hepatocytes grown from iPSCs, and after implanting it in a polycaprolactone scaffold, it was successful when human albumin was found in the serum of two animal models with tyrosinemia. The most sophisticated human brain circuit system yet built from human skin cells was created in a recent experiment that first transplanted these organoids into young rat brains. This work also showed that implanted human neurons may affect animal behavior [140]. Although organoids have had some success in the field of organ transplantation, there are still several obstacles that need to be overcome in order to attain widespread clinical applications. These obstacles include organoid size, cell maturation, transplantation locations, and immunological rejection. Organoids can mimic part of the organ structure and function, but they usually fail to achieve the complexity and functional integrity comparable to that of mature organs. Examples include incomplete cell differentiation, simplified tissue architecture, and lack of innervation and immune cell distribution. And the immune rejection faced after organoid transplantation is also a significant challenge. Lack of mature immunoregulatory mechanisms may lead to graft failure, and there is still a need to develop effective immunosuppressive strategies or techniques for inducing immune tolerance [141].

## 5 Limitations

There are several limitations to this study. To begin, just one database, Web of Science Core Collection, was chosen for investigation, with other significant medical databases like PubMed, Scopus, and EMBASE being omitted. Second, the study may have been underrepresented due to the restrictions on language type, publication kind, and time period. Also, due to the ongoing upgrading of databases, some of the most current papers may not appear in our analysis. As a result, there may be some discrepancy between the study findings and the actual situation.

## 6 Conclusions

Through the bibliometric analysis of global organoid research over the past 22 years, we reveal that the research hotspots in this field are focused on four major topics: personalized medicine, disease modelling, gene repair, and regenerative medicine, which are not only of great interest at present but are also expected to be the dominant trends in the future. This research's results will help academics clearly understand the core issues and boundaries of organoid research and provide strong guidance for researchers to plan their future research paths. To further promote the deepening and innovation of organoid research, we need to optimize and standardize the function of organoids by improving the culture strategy, simulating the microenvironment, and applying bioengineering technology to enhance the accuracy and stability of organoids to more closely match the physiological characteristics of natural organs and improve the reproducibility of experiments. In addition, it is also necessary to promote the practical application of organoids in drug screening, personalized medicine, and regenerative medicine and to study the technical bottlenecks in the large-scale production, storage, and transportation of organoids so as to accelerate the pace of organoid technology from the laboratory to the clinic and the market. In terms of cooperation, it is necessary to strengthen the in-depth collaboration between universities, research institutes, companies, and medical institutions in various countries to promote the practical application of organoid technology jointly. On the other hand, it is necessary to advocate the openness and sharing of data in organoid research and establish open databases and online platforms to facilitate researchers' access to, contribution to, and analysis of data so as to promote the rapid development of the whole field.

Supplementary Materials: The following supporting information can be downloaded at: www.mdpi.com/xxx/s1, Figs. S1–S3.

## Funding

This work was supported by 10.13039/501100003785Medical Research Foundation of Guangdong Province (No.A2022362), the Scientific Research Projects of Medical and Health Institutions of Longhua District，Shenzhen (No.2021017), Shenzhen Science and Technology Planning Project (No.JCYJ20220530165014033) , Medical research project of Shenzhen Longhua Medical Association (2023LHMA10) and Shenzhen Medical Key Discipline (No.MKD202007090201).

## Data availability statement

The data for this study are all sourced from WOS databases, and the original contributions are included in the article/supplementary materials.

## Ethics approval and consent to participate

No applicable.

## Consent for publication

No applicable.

## CRediT authorship contribution statement

**Yantong Wan:** Writing – review & editing, Writing – original draft, Formal analysis, Data curation, Conceptualization. **Jianan Ding:** Writing – review & editing, Writing – original draft, Validation, Formal analysis, Data curation, Conceptualization. **Zixuan Jia:** Writing – review & editing, Validation. **Yinghao Hong:** Writing – review & editing, Writing – original draft, Formal analysis, Data curation, Conceptualization. **Guijie Tian:** Writing – review & editing, Validation. **Shuqian Zheng:** Writing – review & editing, Validation. **Pinfei Pan:** Writing – review & editing, Validation. **Jieyan Wang:** Writing – review & editing, Validation. **Hui Liang:** Writing – review & editing, Validation.

## Declaration of competing interest

The authors declare that they have no known competing financial interests or personal relationships that could have appeared to influence the work reported in this paper.

## Figures and Tables

**figs1:**
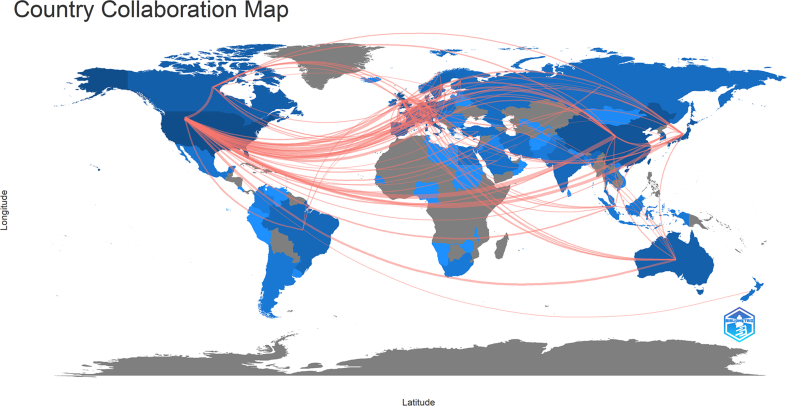


**figs2:**
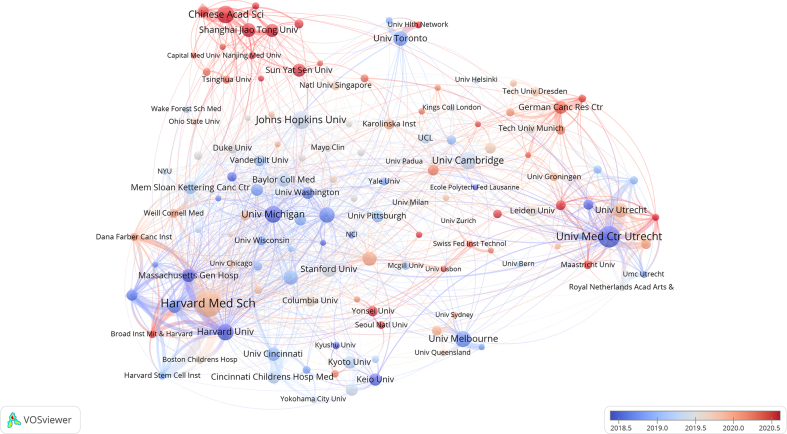


**figs3:**
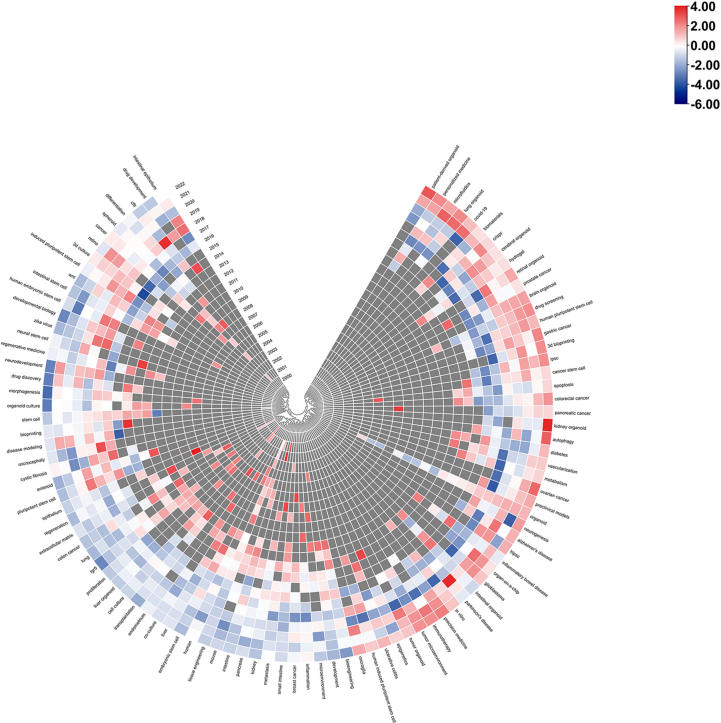


**figs4:**